# Measuring the Impacts of Extra-Musical Elements in Guitar Music Playing: A Pilot Study

**DOI:** 10.3389/fpsyg.2020.01964

**Published:** 2020-08-14

**Authors:** Isabelle Héroux, Sergio Giraldo, Rafael Ramírez, Francis Dubé, Andrea Creech, Louis-Édouard Thouin-Poppe

**Affiliations:** ^1^Department of Music, Faculty of Art, University of Quebec in Montreal, Montreal, QC, Canada; ^2^Music and Machine Learning Lab, Music Technology Group, Pompeu Fabra University, Barcelona, Spain; ^3^Schulich School of Music, McGill University, Montreal, QC, Canada

**Keywords:** expressive performance, music interpretation, methodology, creativity, guitar evaluation, audio recording, audio data analysis, extra musical element

## Abstract

Philosophers, composers, and musicians have long argued whether instrumental music finds meaning in its formal structure and musical content (Hanslick, 1986) or through reference to extra-musical elements, like narratives, emotions, or memories (Meyer, 1956). While the use of extra-musical elements appears grounded in individual musicians’ priorities for performance and teaching (Héroux, 2018), the impact of emotional indications on expressivity has not previously been studied in a large-scale experiment. The aim of this pilot study was to construct the methodology for a larger project to study the impact of the use of extra-musical elements on the sound results of guitarists. We asked guitar students to record one short newly composed piece, Evocation 1, according to the following conditions: (A) in a non-expressive manner, (B) according to the notated musical indications, and (C) with the addition of suggested contextual and emotional extra-musical elements to the musical instructions. We asked two expert guitarists to evaluate the level of expressiveness for conditions B and C and conducted interviews with participants to collect data on the experimental process to refine protocol. To more objectively measure manifestations of objectivity from the recorded performances, we extracted data from each recording about pitch, dynamics, and timing, as well as expressive dynamic deviations. The impact of both recording conditions and the expertise level of performers on the quality of this audio data led us to change the analysis design from a comparative design (with other participants) to a self-comparative design (each participant with himself) for the larger study.

## Introduction

For some scholars of interdisciplinary research on artistic creativity (e.g., Lubart and Getz, 1997; Lubart et al., 2015), making analogies is an essential element of the creative process. The analogies are emotional or cognitive associations between an idea or a task to perform (here, interpreting a musical annotation) and lived or fictional elements that do not have direct links to the task. For Lubart et al. (2015), analogies can be made with images, memories, and narratives. Making analogies is essential to the construction of metaphors frequently used in descriptions and accounts of musical processes (Héroux and Fortier, 2014). It is also common for teachers to use analogies to illustrate musical ideas (Tait, 1992; Hastings, 2006; Meissner, 2016); for example, asking a student to play a long crescendo as if it was a sunrise. However, philosophers of music, composers, and performers have argued for decades whether music finds meaning through its form and in the objective musical material (e.g., Hanslick, 1986) or through reference to extra-musical elements (e.g., Meyer, 1956). In a recent analysis of the creative process of interpretation from nine professional musicians, Héroux (2018) identified the same “debate,” which appears grounded in the musicians’ artistic approaches, working habits, values, beliefs, or priorities. In her study, the majority of experts used extra-musical elements. A minority chose not to do so, considering this an approach that does not respect « the indications of the musical text and the composer’s intentions » and which compromises the authenticity of the work of art (Héroux, p. 12). If the choice to use or not use extra-musical elements is based on musicians’ approaches, this choice could also significantly influence the expressiveness of their playing. Prior research has demonstrated that the use of extra-musical elements can impact the musician’s subjective experience during performance in order to develop a more expressively accurate feeling during the artistic appropriation phase, which is characterized by “a quest for feeling of expressive precision and balance in the playing and by an exploration of musical character, nuance, sonority, phrasing, etc.” (Héroux, 2016, p. 307). Unfortunately, we still lack both a means of identifying the extent of this impact on the expressivity and measuring it accurately. To explore this phenomenon more objectively, we will use a quantitative approach to measure the influence of the use of extra-musical elements on the sound results of expert guitarists. To do so, this research aims to pilot one possible quantitative methodological approach realized with one musical piece (M1) to measure the use and impact of extra-musical elements on the musical expressiveness of a small sample of guitarists. To achieve this objective, a main question was formulated: How could we objectively and subjectively measure the use of extra-musical elements in the instrumental playing of guitarists? This led to three sub-questions of a methodological nature: (1) which parameters should guide the composition of the work to be used in this experimentation, (2) how can we measure expert musicians’ perceptions of the expressivity of the guitarists’ playing? and (3) how can we measure acoustic changes in the sound results, whether or not the extra-musical elements are used? This article presents the methodological approach explored with this pilot using a work composed for the study as well as the decisions taken, following the results obtained, to conduct a larger research project with 90 expert guitarists. We asked four undergraduate-level guitarists and one graduate-level guitarist to each make two recordings of the piece according to three conditions: (A) in a non-expressive manner, (B) according to the musical indications’ instructions written in the score, and (C) with the addition of extra-musical elements (contextual and emotional) to the musical instructions suggested on the score. Condition A was administered first to allow the musicians to become familiar with the work, and conditions B and C were randomly assigned. We then asked two professional guitarists with more than 20 years of experience as performers, guitar teachers at the university level and music competition adjudicators, to evaluate the level of expressiveness and the level of overall appreciation for conditions B and C. Following this stage, we next conducted interviews with participants to obtain feedback on the experimental protocol including evaluation process. In addition, to measure manifestations of expressivity objectively from these performances, we extracted auditory data from each recording about pitch, dynamics and, timing, as well as expressive dynamics and deviations. From these results, we used machine learning techniques to classify interpretations into conditions A, B, and C. In addition, we conducted post-task interviews with the two expert musicians to get their feedback on the methodology and evaluation processes.

## Materials and Methods

**FIGURE 1:**
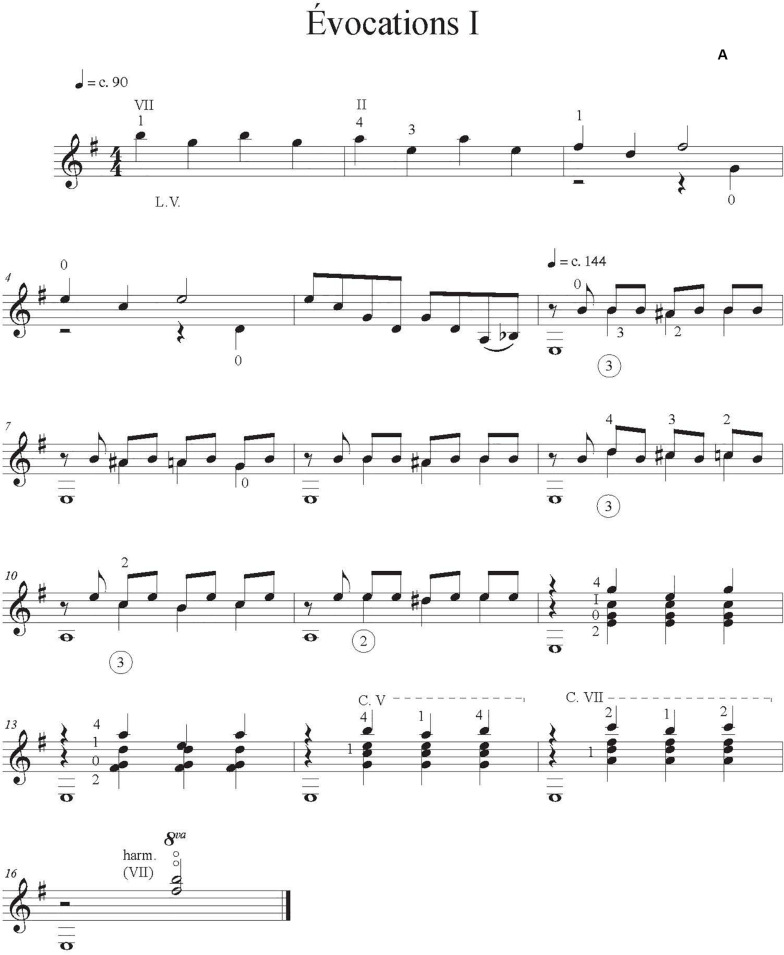
Score condition with only the notes, rhythm, and basic tempo indications.

**FIGURE 2:**
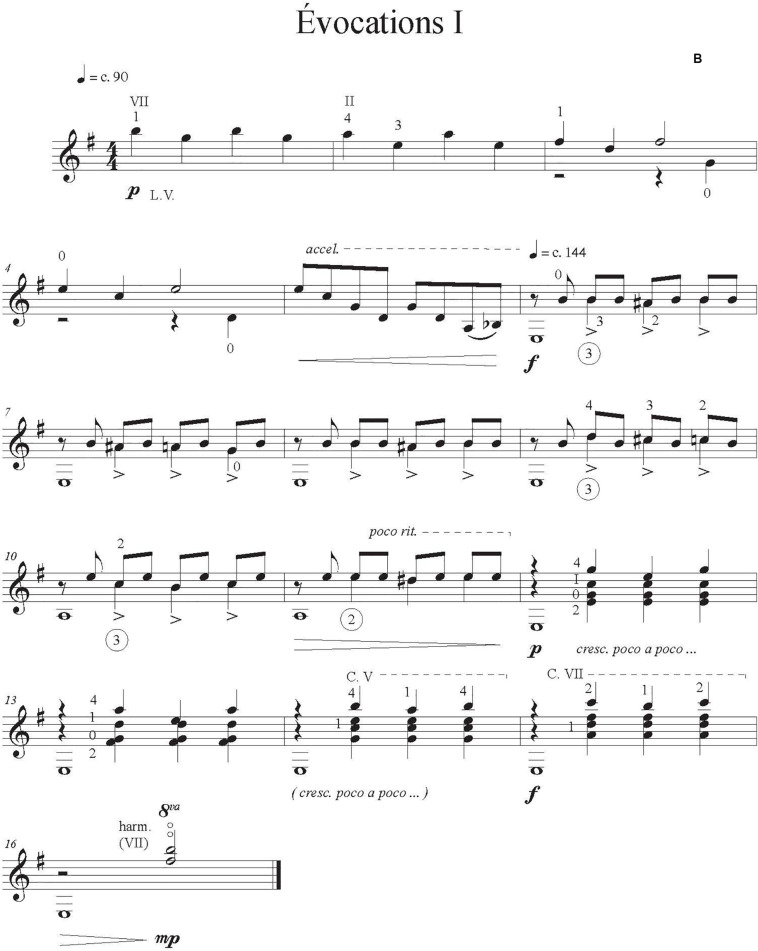
Score condition B with more detailed performance indications.

**FIGURE 3:**
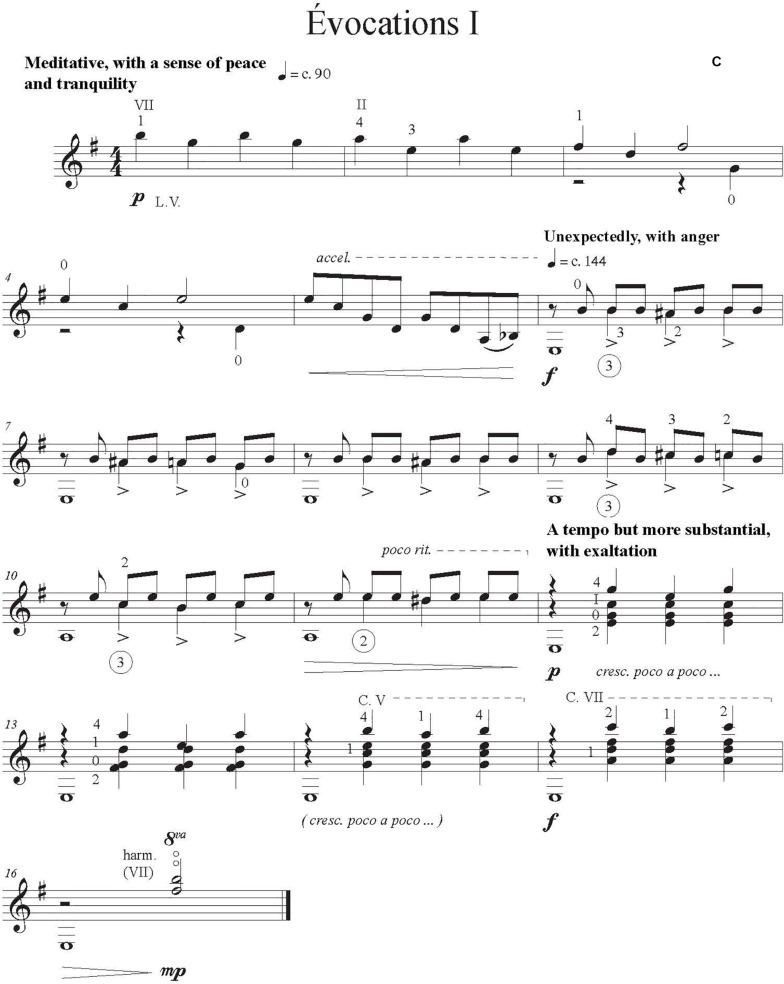
Score condition C one annotated with the intended emotions as well as all of the indications provided in B.

As test works for our experiment, we asked professional composer and guitarist Jimmie Leblanc to create a short and easy original solo guitar work of about 30 s. We instructed him to include in the composition at least two (a) contrasting sections, (b) character indications, (c) dynamic indications, and (d) emotions evoked by the music and written in the score. We provided a list of music emotions from Zentner et al. (2008) extracted from four studies (1392 participants). These are divided into three contrasting categories: *sublimity* (i.e., nostalgia); *vitality* (i.e., heroic); and *unease* (i.e., sad). We asked the composer to include at least one emotion from two different categories. Indications of attacks and changes of timbre were at the composers’ discretion. He also provided three scores that corresponded to our three conditions: (A) one with only the notes, rhythm, and basic tempo indications (metronomic marks), Figure 1; (B) one that had more detailed performance indications (tempo variation, dynamics), Figure 2; and (C) one annotated with the intended emotions as well as all of the indications provided in B, Figure 3.

### Recording Process

The musicians used their own guitar for the recordings, in order to keep the experimentation as natural as possible. Guitarists, in contrast to pianists, are used to playing with individual instruments that vary in specifications (size, sound, strings, etc.), and changing instruments could be an obstacle to expressivity in such a short time to record the music.

As the final project aims to record 90 participants, we decided to use portable studio equipment that would allow us to record in different locations with similar conditions: *HP* laptop with *Pro-Tools* recording software, audio interface (*Focusrite: Scarlett 2i2*) and a clip-on microphone for guitar (*DPA 4099* instrument microphone).

The pilot study took place in Université du Québec à Montréal’s Music Department recording studio, between June and August 2018, with four undergraduate guitar students. We provided them three scores of the same composition as described in the methodology section. Each participant had 15 min to practice condition A before recording it. The score for B or C was provided to them when they finished recording A. We assigned an identification number to all musicians. The guitarists with an odd identification numbers (e.g., 1, 3) received condition B followed by C. Those with even identification numbers received condition C followed by B. We gave 15 min to the participants to practice and record each condition for a maximum experimentation duration of 45 min. Participants could record as many takes of a condition as they wished, so long as they respected the 15 min allowed for each condition. Participants then selected their preferred take for each condition. The researchers selected a second recording for each participant in order to double the data to test the experts’ evaluation and audio analysis processes. Following this stage, participants completed a post-recording questionnaire on SurveyGizmo about their academic and professional background, their own interpretative approach, and the approaches used during the study.

This information was collected only to test the administration of the questionnaire, the online platform, and the clarity of the questions for our eventual final study, for which the pilot undergraduate participants would not qualify. In post-task semi-structured interviews, we also collected participants’ thoughts on the experiment process (e.g., appropriateness of time allowed, music to perform). We ended the recording process with four participants and eight recordings (S01–S08).

### Recording Evaluations and Interviews

We asked two expert guitarists, E1 and E2, to blindly evaluate all recordings of conditions B and C. They had (respectively) 20 and 30 years of experience as concert artists, college and university guitar teachers, and jury members for international guitar competitions. Evaluations took place from July to September 2018 and were realized with *Google Form* questions with integrated audio tracks. For each recording, they answered two questions on a Likert scale from 1 (least) to 6 (most) about (A) the perceived level of expressiveness of the musical interpretation and (B) their level of overall appreciation. We analyzed data with simple descriptive statistics on Excel (means, correlations). We conducted a post-task semi-structured interview with them on the 16th of July 2018 about the evaluation process, general comments, and further suggestions.

### Audio Data Analysis

The musical composition used for the recordings was polyphonic, i.e., having two or more notes played at the same time, though not necessarily with the same duration(s). A maximum of four-note polyphony was considered. The score was provided in both MIDI and Music xml. Both formats encoded the information of each note with respect to its onset, duration, pitch, and velocity (volume). Xml format permitted us to encode other relevant score annotations, such as time signatures, key, chords (not used in this study), and lyrics. This information was later used to extract descriptors from the score.

**FIGURE 4:**
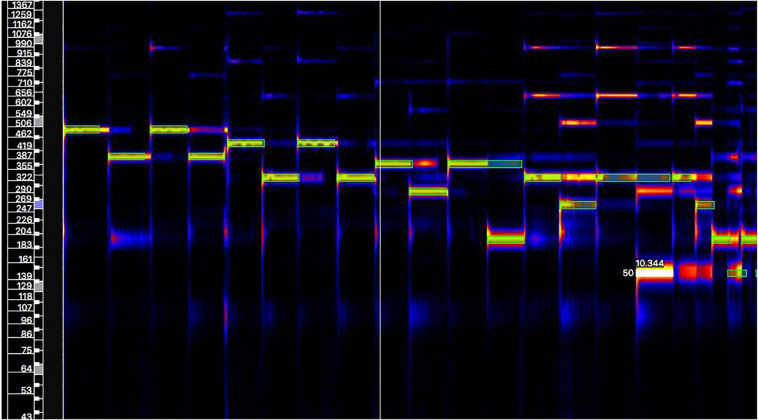
Visualization of the spectrogram and the manually corrected transcription (green boxes).

For data processing, we followed a similar approach used in prior research (Giraldo and Ramirez, 2016a, b). Semiautomatic audio transcription from the recordings was performed to convert information on each note of the recorded performance into a machine-readable format, i.e., in terms of its duration, onset time, pitch, and velocity (dynamics/volume). To do so, non-negative matrix factorization (NMF) was used (Benetos et al., 2012). In this technique, the spectrogram is decomposed into an activation matrix H, which represents the energy (dynamic level) of each pitch bin (i.e., the number of different pitches present in the score) over time, and a matrix W containing the spectral basis, which represents the energy of the harmonic components of each pitch bin over frequency. Score information and spectral information (i.e., the spectral analysis of individual notes samples of a classical nylon guitar) were used to initialize both the activation matrix and the spectral basis. Manual correction was performed to eliminate the automatic transcription errors. For this purpose, we visually compared the obtained transcription with the spectrogram (see Figure 4), using a visualization tool (Cannam et al., 2010). We discarded incorrect notes from both the automatic transcription (i.e., notes that differ from the score) and the performance (i.e., errors on the actual performance). A total of 54 guitar performances were analyzed, resulting in 3046 notes.

#### Expressive Performance Actions

Expressive performance actions are the performance deviations (with respect to the score) introduced by the performer consciously (e.g., to convey an intended emotion) or unconsciously (e.g., reflecting the musical background or preferred style of the performer). In order to obtain representative performance actions, we compared the computer-readable information of the score and the transcription obtained from each of the considered performances. A data set for each performance and each performer was created in which each note is represented by its pitch, duration, onset, and energy along with its computed deviations. The performance actions considered for this study are the following.

##### Timing deviation

We performed automatic beat detection using multifeature beat tracking (Zapata et al., 2014), and post-processed the output by manual correction. A complication arises at this stage, given that both musical pieces have tempo changes, i.e., they consist of two sections with different tempi, i.e., beat per minute (BPM). Therefore, the automatic beat tracking process outputs significant errors, making it necessary to perform manual correction.

##### Duration deviation

Inter-onset interval (IOI) is the difference between the onset of two consecutive notes, which may be used for measuring note duration. On the guitar, the duration of the note is generally governed by the natural decay of the vibration of the string. We consider IOI an accurate strategy for measuring notes’ duration differences. Thus, for each note, we measured the difference between the IOI of a note in the score and its corresponding note in the performance.

##### Energy (dynamics) deviation

Based on the note segmentation performed at the automatic note transcription, this task was performed on the IOI of each note by measuring the root mean square (RMS) value of the corresponding audio signal segment. Polyphonic sections (i.e., chords) were treated as monophonic ones. Here we assume that the total energy of a performed chord is the sum of the energy of notes of the chord, in which each note provides the amount of energy which was estimated from the activation matrix obtained at the NMF stage. The RMS level was normalized to MIDI velocity values (from 1 to 127), and the energy deviation is obtained by calculating the difference of the score velocity of each note in the score (set to 64 by default), and the corresponding performed note.

#### Correlation Measurements

**FIGURE 5:**
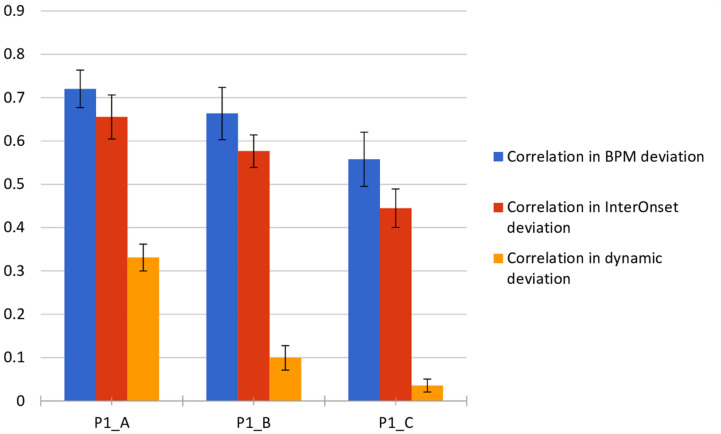
Correlation mean and standard deviation among performers for each interpretation type (A, B, C) and performance action, at the musical piece considered.

For each note in the performances, we obtained a deviation measure for the three performance actions considered. Each performance was treated as a separate dataset with the same number of instances (i.e., notes). The sequences of values of the performance actions were treated as numerical series, for which we computed the correlation among the variations introduced by the performers for each type of performance, being A: inexpressive, B: with score expressive indications, and C: expressive/imaginary. We computed the correlation for each performance action and performance type, using a one vs. all strategy among performers, obtaining correlation matrices (see the spreadsheet in the Supplementary Material section). We then computed the mean and the average of the obtained correlation values at each matrix, for which the summarized results are presented in Figure 5.

## Results

### Experts Recording Evaluations and Processes

Experts considered condition B more expressive, and condition B was more appreciated than C. Preliminary results indicate that the induced computational models were able to classify with a mean average accuracy of 87% (well above the 33% the baseline) the expressiveness of conditions B and C.

In the post-task interviews, the experts mention that the technical weaknesses that participants demonstrated in the recordings made it almost impossible to evaluate the expressiveness of the playing and impacted their appreciation of the playing for each condition: “I thought to myself, ‘Why didn’t they take people who…played the excerpts better, who knew them better and who […] have made it possible to evaluate expressiveness on a more solid basis.”’ E1: (6:32).

(a) The experts did not agree on the benefit of having the score for evaluation, and (b) they found the instructions and the process easy and were able to evaluate the two pilot recordings (*n* = 18) in ± 60 min.

For their part, undergraduate student participants told us they wished to have more time to practice in preparation for the recordings. We decided to allow a maximum of 20 min of practice before each condition’s recording.

### Audio Recording Analysis

Results, summarized in Figure 2, show correlations between mean and standard deviation among performers for each interpretative condition (A, B, C) and performance action. The Supplementary Material section provides all results for each participant in an Excel spreadsheet. In this pilot, a tendency of correlation among performances was observed in which we found higher levels of correlation in tempo variations. Next levels of correlation were obtained on IOI differences, which are closely related to BPM changes. Finally, the lowest correlation obtained, with higher standard deviation values, was obtained for note energy, which indicates a high degree of randomness.

## Discussion

The aim of this pilot was to develop a methodology to study, with objective and subjective measurements, the impact of extra-musical elements on the musician’s expressivity in a larger project. We experimented with one original composition. Student participants were instructed to practice and record the music following three conditions: (A) with only the notes, rhythm, and basic tempo marks; (B) with musical indications (tempo variation, dynamics); and (C) with emotions evoked in addition to all the indications that were provided in A and B conditions. We then asked two expert-level guitarists to evaluate, based on a Likert scale, the expressivity of the and their appreciation interpretation of conditions B and C for the pilot study.

To measure acoustic changes in the sound result depending on whether or not the extra-musical elements are used, we extracted acoustic data from the recordings of conditions A, B, and C and proceeded to correlation analysis. This specific question led to methodological challenges: quality of audio data uniformity of recording conditions and the expertise of performers, which leads us to now change the analysis design moving forward.

### The Audio Data

The accuracy of the obtained computational models indicates that the deviations performed by the musicians, as well as the features extracted from the audio recordings, contain sufficient information for the model to discriminate among performances in conditions A, B, and C. However, mistakes or technical difficulties experienced by participants made the task of evaluating the recordings difficult for the experts in the evaluation process. These non-expressive aspects of the performances may have also affected the induced computational models. It was possible to spot minor errors (e.g., wrong notes) to modify them; however, there were cases in which the performer erroneously repeated the same bar/section of the piece. These types of errors must be avoided for the sake of automatic transcription from the audio informed by the score, which assumes that the number of performed notes is equal to those notated in the score. The alignment of the score and the performance is a critical step in obtaining this transcription. If the performer repeats a small section due to a mistake, it has a huge impact on the error at the automatic alignment and on the consecutive analysis of the data. In this sense, the choice of music and the level of expertise of musicians are crucial to conducting the main research project to help musicians to concentrate on the expression (rather than the accuracy) of their performances.

This pilot study was conducted with students to allow us to fine-tune the experimentation procedures and analysis. As the large-scale experiment will be conducted with only with graduate- and professional-level guitarists, the quality of performances should improve. Moreover, we will tell musicians when they make mistakes on a recording take to help them to correct it. Participants will now be allowed 20 min for each condition.

We will also consider recording more than 90 musicians, in order to have at least 270 recordings without any repeated bars or notes. The number of participants will allow us to segment the data in order to determine whether the order in which the conditions are recorded will have an impact on the results (B and C or C and B). If this is the case, we will keep only the participants who recorded condition B before condition C and still have 45 participants (135 recordings) to perform the analyses.

### Correlation Analysis

The higher levels of correlation in terms of variations in tempo might be biased by indicated tempo changes in the score (bar 6), which all of the performers followed. As IOI differences are closely related to BPM changes, however, because the IOI differences were obtained on a note-level basis, this might be a more realistic measure in which the bias introduced by the tempo changes in the piece might have less influence. For the lower correlation obtained, higher standard deviation values were obtained for note energy, which indicates a high degree of randomness. This might be due to differences in the acoustic conditions at the recording stage (position of the microphones), differences in their instrument’s qualities (i.e., the individual guitars utilized), and individual technique.

### Changes in Analysis Design

In order to obtain better indicators of dynamics and spectral features across a variety of rooms, instruments, and microphone setups, we decided to change the analysis design. Instead of obtaining correlation matrices of each participant in each condition to the others, we will proceed first to an individual measurement of the criteria for each participant for each condition. First, we will measure deviations between A and B and between A and C, so that we can determine factors of deviance for each participant individually. In this design, each participant will be compared with themselves to avoid differences in recording conditions. Secondly, we will compare the factor of deviancy for the criteria between B and C for all participants. With this approach, we will be able to measure more accurately which condition, between B or C, is the most «expressive» for all participants, by evaluating the level of correlation between the mean and the average of deviance from condition A–B and A–C of all participants.

For the large-scale experiment, we plan to follow a similar approach to estimate the RMS values at each IOI, extracting values for other relevant spectral features, e.g., kurtosis, skewness, tristimulus, and MFCCs, which are indicators of timbre quality. Timbre is a relevant performance action that might be used by the performers by manipulating the finger-string attack.

Another stage will use machine learning techniques to extract features from the notes in the score and train models to predict the performance actions introduced by the performers. We will evaluate the correlation of the predictions and the actual deviations introduced by the performers, which could help us determine whether the score contains sufficient information to induce these performance actions.

For our further research, we also aim to compare recording analysis correlation results with subjective evaluations made by two experts. At this stage, we will observe whether there is a correlation between the perceived degree of expressiveness, the instruction applied by the interpreter, and the variation in the measured acoustic parameters.

## Data Availability Statement

All datasets generated for this study are included in the article/Supplementary Material.

## Ethics Statement

This study was carried out in accordance with the recommendations of the Politique no 54 sur l’éthique de la recherche avec des êtres humains (December 2015). All subjects gave written informed consent in accordance with the Declaration of Helsinki. The protocol was approved by the Comite institutionnel d’éthique de la recherche avec des êtres humains de l’Université du Québec à Montréal (Certificat 1960_e_2017). The participants provided their written informed consent to participate in this study. Written informed consent was obtained from the individuals in the study for the publication of any potentially identifiable images or data included in this article.

## Author Contributions

IH, FD, and AC contributed to the conception and design of the study. IH contributed to the realization of the experimentations and wrote the first draft of the other parts with FD. SG and RR designed the study. SG also performed the statistical analysis and wrote related sections of the manuscript. L-ÉT-P conducted the evaluation task with the two experts and associated data analysis. All authors contributed to manuscript revision and read and approved the final submitted version.

## Conflict of Interest

The authors declare that the research was conducted in the absence of any commercial or financial relationships that could be construed as a potential conflict of interest.

## References

[B1] Benetos E. Dixon S. Giannoulis D. Kirchhoff H. Klapuri A. (2012). Automatic music transcription: breaking the glass ceiling, *Paper presented at the 13th International Society for Music Information Retrieval Conference (ISMIR 2012)*, Portugal.

[B2] Cannam C. Landone C. Sandler M. (2010). “Sonic visualiser: an open source application for viewing, analysing, and annotating music audio files,” in *Proceedings of the 18th ACM international Conference on Multimedia*, (New York, NY: ACM), 1467–1468. 10.1145/1873951.1874248

[B3] Giraldo S. I. Ramirez R. (2016a). A machine learning approach to discover rules for expressive performance actions in jazz guitar music. *Front. Psychol.* 7:1965. 10.3389/fpsyg.2016.01965 28066290 PMC5167744

[B4] Giraldo S. Ramirez R. (2016b). A machine learning approach to ornamentation modeling and synthesis in jazz guitar. *J. Math. Music* 10 107–126. 10.1080/17459737.2016.1207814

[B5] Hanslick E. (1986). *Du Beau Dans la Musique.* Trans. C. Bannelier. Paris: Christian Bourgeois.

[B6] Hastings C. (2006). *The performer’s Role: Storytelling in Ballades of Chopin and Brahms*, PhD Thesis, Ann Arbor, MI: University of Michigan.

[B7] Héroux I. (2016). Understanding the creative process in the shaping of an interpretation by expert musicians: two case studies. *Music. Sci.* 20 304–324. 10.1177/1029864916634422

[B8] Héroux I. (2018). Creative processes in the shaping of a musical interpretation: a study of nine professional musicians. *Front. Psychol.* 9 665. 10.3389/fpsyg.2018.00665 29867643 PMC5952184

[B9] Héroux I. Fortier M.-S. (2014). Expérimentation d’une méthodologie pour expliciter le processus de création d’une interprétation musicale. *Les cahiers de la Société québécoise de recherche en musique* 15 67–79. 10.7202/1033796ar

[B10] Lubart T. I. Getz I. (1997). Emotion, metaphor, and the creative process. *Creat. Res. J.* 10 285–301. 10.1207/s15326934crj1004_1

[B11] Lubart T. I. Mouchiroud C. Tordjman S. Zenasni F. (2015). *Psychologie de la Créativité*, 2nd Edn Paris: Armand Colin.

[B12] Meissner H. (2016). Instrumental teachers’ instructional strategies for facilitating children’s learning of expressive music performance: an exploratory study. *Inter. J. Music Educ.* 35 118–135. 10.1177/0255761416643850

[B13] Meyer L. B. (1956). *Emotion and Meaning in Music.* Chicago: Chicago University Press.

[B14] Tait M. J. (1992). “Teaching strategies and styles,” in *Handbook of Research on Music Teaching and Learning*, ed. Colwell R. (New York, NY: Schirmer Books), 525–534.

[B15] Zapata J. R. Davies M. E. Gómez E. (2014). Multi-feature beat tracking. *IEEE/ACM Trans. Audio Speech and Lang. Process.* 22 816–825. 10.1109/TASLP.2014.2305252

[B16] Zentner M. Grandjean D. Scherer K. R. (2008). Emotions evoked by the sound of music: Characterization, classification, and measurement. *Emotion* 8 494–521. 10.1037/1528-3542.8.4.494 18729581

